# Loss of the starvation-and-light fruitbody formation trigger in the myxomycete *Physarum roseum*

**DOI:** 10.1098/rsbl.2025.0215

**Published:** 2025-07-30

**Authors:** Mana Masui, Phillip K. Yamamoto, Nobuaki Kono

**Affiliations:** ^1^ Graduate School of Media and Governance, Keio University, Fujisawa, Kanagawa Prefecture, Japan; ^2^ Institute for Advanced Biosciences, Keio University, Tsuruoka, Yamagata Prefecture, Japan; ^3^ Faculty of Environment and Information Studies, Keio University, Fujisawa, Kanagawa Prefecture, Japan

**Keywords:** slime mould, myxomycetes, plasmodium, sporulation, RNA-seq

## Abstract

Myxomycetes are unicellular amoebozoans that form fruiting bodies to reproduce, a process known as sporulation. In the model species *Physarum polycephalum*, plasmodia form fruiting bodies only after several days of starvation followed by light exposure. It has long been assumed that the same starvation-plus-light trigger applies to the genus *Physarum*. Recent observations of congeners that fail to sporulate under the same conditions have raised doubts about this assumption and prompted tentative taxonomic reconsideration. Because comparable starvation and light tests are rare for other species of *Physarum*, their phenotypes and molecular mechanisms remain unclear. Consequently, we investigated *Physarum rigidum* and *Physarum roseum* under starvation and light conditions. Four of the six *P. rigidum* plasmodia sporulated by day 6, whereas *P. roseum* did not sporulate within 7 days. RNA-seq of *P. roseum* across nutrient-rich/starved and dark/light conditions revealed differential expression was driven chiefly by nutrition; light caused only minor changes and did not elicit the transcriptional programme characteristic of *P. polycephalum* sporulation. The photoreceptor genes that drive sporulation in *P. polycephalum* were not detected in *P. roseum*, and 92 candidate photoreceptor genes showed no significant regulation. These findings suggest that *P. roseum* responds only minimally to light stimulation and that the starvation-plus-light trigger is not universally retained within the genus *Physarum*.

## 1. Introduction

Myxomycetes (slime moulds) are multinucleate unicellular organisms belonging to Amoebozoa and are known to display diverse morphologies throughout their life cycle [1]. The plasmodium moves at approximately 1 cm h^−1^ [2] and typically inhabits moist, dark microhabitats such as decaying wood or leaf litter, where it preys on bacteria and fungi [3]. When exposed to environmental stressors such as starvation or light, the plasmodium responds either by entering a metabolically inactive dormant state (sclerotium) or by forming fruiting bodies. In the model, myxomycetes *Physarum polycephalum* and *Didymium iridis*, starvation and light have been demonstrated to be indispensable triggers for sporulation [4,5]. After more than 3 days of preconditioning by starvation, *P. polycephalum* synthesizes several photoreceptors, including phytochrome-like proteins that respond to wavelengths from the visible to the near-infrared range. A brief far-red pulse (approx. 700 nm) or several hours of continuous visible light then acts as a trigger, inducing sporulation [6,7]. Although plasmodia generally exhibit negative phototaxis [8], starvation can occasionally induce positive phototactic behaviour [9]. In the absence of light stimulation following starvation, the plasmodium bypasses sporulation and instead forms a sclerotium [10,11].

Fruiting-body induction by combined starvation and light stimuli is thought to be widely conserved across species of the genus *Physarum* [12,13]. However, the traditional genus *Physarum* is clearly monophyletic; the similarity of many species is a result of convergent evolution [14,15]. Members of this genus also share core ecological traits, including similar microhabitats, a large phaneroplasmodium and prolific fruiting [16], and they are widely employed as model organisms in myxomycetes research. Recent molecular phylogenies, however, propose transferring several species formerly assigned to *Physarum* to other genera, reinforcing the growing view that the genus *Physarum* is not monophyletic [14]. Phenotypic data likewise suggest the same pattern. For example, *Physarum nivale* grows only within an exceptionally narrow, low-temperature range [17], whereas our observations show that *Physarum roseum* thrives over a markedly broader temperature range. In the interplasmodial allorecognition (fusion and avoidance) universally observed among myxomycetes, heterospecific fusion is typically absent. In heterospecific encounters within the genus *Physarum*, not only is avoidance behaviour lacking, but the two plasmodia appear unable to recognize each other [18]. Critically, the sporulation trigger may differ among *Physarum* species: *P. roseum* almost never forms fruiting bodies under conditions that reliably induce sporulation in *P. polycephalum* (starvation ≥3 days under dim natural light) [18].

Taken together, these findings suggest that species traditionally assigned to within the genus *Physarum* under classical taxonomy differ markedly in their molecular phylogeny, as well as in their ecology, physiology and cultivation characteristics. Particularly, sporulation is the life cycle stage under the strongest selective pressure, dissecting its interspecific variation is fundamental to understanding evolution within the genus. Nonetheless, detailed ecological, functional and molecular investigations of species of *Physarum* other than *P. polycephalum* are scarce, and their transcriptional responses to starvation and light are virtually unexplored.

To clarify the diversity of responses to the starvation and light cues that induce sporulation, we compared both phenotypic and intracellular reactions in plasmodia of *Physarum* species that are likely to exhibit phenotypes distinct from *P. polycephalum*. Phenotypically, we examined the behavioural responses of these species to starvation and light stimulation. Intracellular dynamics were assessed by differential gene expression analysis: plasmodia were cultured under factorial combinations of light versus dark and nutrient-rich versus starvation conditions, and transcriptomic changes across treatments were profiled by RNA-seq.

## 2. Methods

### (a) Samples

Plasmodia of *P. roseum* strain Ro1 were collected in 2018 during fieldwork at the Midori-no-Mori Museum, Saitama Prefecture, Japan, and plasmodia of *Physarum rigidum* strain Ri1 were obtained from the same place in 2020. Both strains had already been taxonomically verified in a previous study [18] by DNA barcoding of the SSU rRNA (Small subunit ribosomal RNA) region with two primer sets and morphological examination of sporocarp characteristics (peridium, spores and capillitium) and were used herein as pre-identified reference strains.

### (b) Culture conditions

Plasmodia were maintained in 9 cm plastic Petri dishes containing 2% plain (nutrient-free) agar. The dishes were kept in an incubator at 23°C and, under normal maintenance, were completely shaded from light. Each day at 16.00, the agar surface was moistened with sterile water, and rolled oats were supplied as a food source.

For RNA-seq, plasmodia of *P. roseum* and *P. rigidum* were cultured for 3 days under four factorial conditions combining light (dark versus light) and nutrition (nutrient-rich versus starved). The conditions were as follows: dark + starved (DS), light + starved (LS), dark + nutrient rich (DN) and light + nutrient rich (LN).

— For the light treatment, the dishes were exposed to diffuse daylight transmitted through a translucent panel and ambient light-emitting diode (LED) room lighting.
— For the starvation treatment, no oats were provided; only sterile water was added during the daily watering time.

All other parameters (dish size, agar composition, incubation temperature and watering schedule) were identical to the maintenance conditions described above.

### (c) Light- and starvation-response assay in *Physarum roseum* and *Physarum rigidum*

Plasmodia of *P. roseum* and *P. rigidum* were transferred to fresh plates of plain (nutrient-free) agar and cultured for up to 7 days under conditions reported to induce sporulation in *P. polycephalum* [7,19]. Cultures were exposed to indirect sunlight passing through a translucent panel, together with ambient LED room lighting, at a photon flux density (PFD) of approximately < 15 µmol m⁻² s⁻¹ ( 380–780 nm with the spectral distribution shown in electronic supplementary material, figure S1), and were supplied daily with sterile water only. Photographs were taken at regular intervals, and the occurrence of sporulation, sclerotium formation or other morphogenetic changes was recorded for each plasmodium strain.

### (d) Total RNA extraction and sequencing

Total RNA extraction was conducted at 11.00 in accordance with a previous protocols [20,21]. Plasmodial tissue (*ca* 5 mm²) was scraped from nutrient-free agar with a sterile toothpick while avoiding residual oats, then immersed in lysis buffer (6 M guanidinium in 1% Triton X-100). The samples were vortexed for 30 s at room temperature, allowed to rest for 1 min and centrifuged at 16 000*g* for 1 min to remove debris. The supernatant was transferred to a fresh tube, mixed with an equal volume of 100% ethanol and purified with a Direct-zol RNA MicroPrep Kit (Zymo Research) according to the manufacturer’s instructions.

RNA purity was assessed with a NanoDrop spectrophotometer (acceptance criteria: OD260/280 ≥ 1.8, OD260/230 ≥ 1.0). The concentration was measured using a Qubit RNA BR Assay, and integrity was verified on an Agilent TapeStation; only samples with an RNA integrity number (RIN) ≥ 8.0 were processed further. Transcriptome libraries were prepared using the NEBNext Poly(A) mRNA Magnetic Isolation Module and the NEBNext Ultra II Directional RNA Library Prep Kit for Illumina. Paired-end 150 bp sequencing was performed on an Illumina NovaSeq X Plus for de novo transcriptome assembly, single-end 75 bp sequencing on an Illumina NextSeq 500 for gene expression analysis.

### (e) De novo transcriptome assembly

We performed de novo transcriptome assembly with Trinity v. 2.1.1 [22] using read data from two experimental conditions—DN and LS—each cultured for 3 days . Each library was prepared from whole plasmodial tissue.

Assembly quality was assessed with seqkit v. 2.6.1 (N50 statistics) and BUSCO v. 5.2.2 (dataset eukaryota_odb10) for completeness. Potential contamination was evaluated with BlobTools v. 1.1.1: contigs were taxonomically assigned by DIAMOND BLASTX v. 2.1.6 against UniProt reference proteomes, and GC-content plots were examined. Contigs forming distinct GC-content clusters and showing prokaryotic BLAST hits were flagged as putative contaminants and removed manually, and the assembly metrics were recalculated.

Functional annotation was generated by retrieving UniProt accessions from BLASTX-positive contigs and mapping them to protein descriptions and Gene Ontology (GO) terms via the UniProt ID Mapper. For genes whose expression varied markedly and attracted particular attention, we performed domain prediction using InterProScan.

### (f) Differential gene expression analysis

Transcript abundance was quantified as TPM using kallisto v. 0.51.1. Differentially expressed genes (DEGs) were identified with edgeR v. 3.42.4 (false-discovery-rate (FDR) < 0.05 and |log₂FC| > 2). All the statistical analyses were conducted in R (base v. 4.3.3).

## 3. Results

### (a) Light- and starvation-induced sporulation in *Physarum roseum* and *Physarum rigidum*

**Figure 1.**
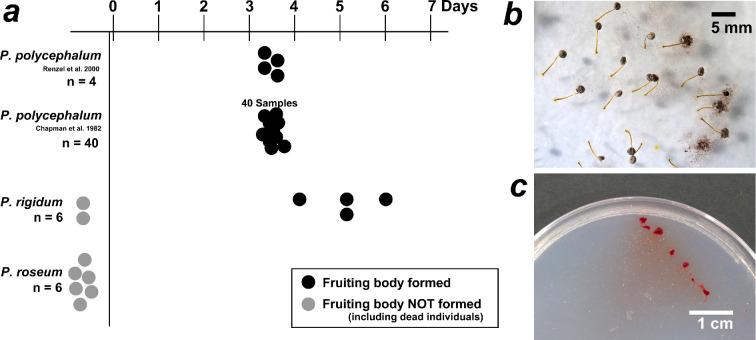
Sporulation test under starvation + light in three *Physarum* species (*a*) The black symbols, plasmodia that sporulated; the grey symbols, those that did not. All *Physarum polycephalum* sporulated by day 3; none of *Physarum roseum* did so in 7 days. About 60% of *Physarum rigidum* sporulated by day 6. (*b*) *Physarum rigidum* sporulating after 4 days; spores surround the fruiting body. (*c*) *Physarum roseum* at day 7, unsporulated, shrunken and presumed dead.

Previous studies have shown that *P. polycephalum* achieved 100% sporulation after 3 days of starvation followed by light exposure (4/4 plasmodia Renzel [7]; 40/40 [19]). With the same regimen, we monitored six plasmodia each of *P. rigidum* and *P. roseum* for 7 days. In *P. rigidum*, four of the six samples sporulated—one on day 4, two on day 5 and one on day 6—while two failed to sporulate (figure 1*a*,*b*). In *P. roseum*, none of the six plasmodia formed fruiting bodies within 7 days, and three specimens died by day 7 (figure 1*a*,*c*).

### (b) De novo transcriptome assembly of *Physarum roseum*

*Physarum roseum*, in contrast to *P. polycephalum* and *P. rigidum*, does not form fruiting bodies in response to starvation and light exposure. Therefore, we focused our transcriptomic analysis on this species, *P. roseum*, alone at this stage. Due to the lack of a genome or transcriptome reference for *P. roseum*, a de novo transcriptome assembly was constructed.

**Figure 2.**
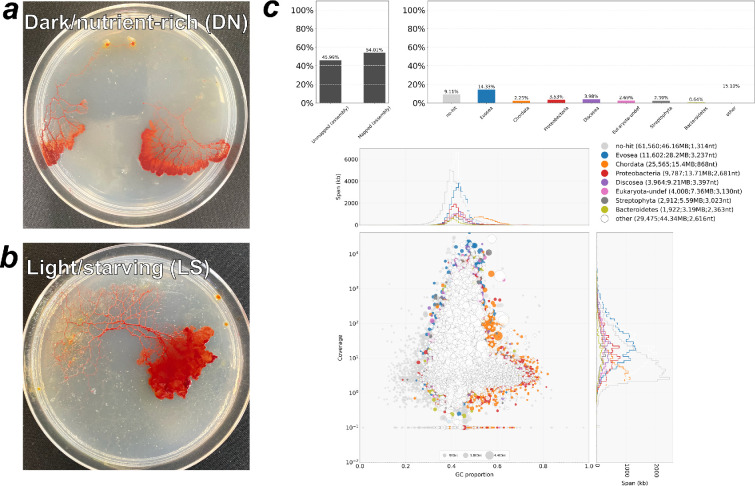
Plasmodium of *Physarum roseum* used to create transcriptome assemblies and confirmation of contamination through data analysis. (*a*) Plasmodium under dark/nutrient-rich (DN) conditions. (*b*) Plasmodium under light/starved (LS) conditions. (*c*) Contamination assessment with BlobTools. The analysis displays the relative abundance of each taxon in the assembly together with their GC content. Hits annotated as Evosea likely represent Myxomycetes, and no-hit contigs with similar GC are also probably myxomycete sequences, not contaminants, due to limited reference data.

Total RNA was extracted from plasmodia cultured under two contrasting conditions: DN and LS (figure 2*a*,*b*). The DN condition represents routine maintenance, whereas LS is its physiological opposite. Paired-end 150 bp sequencing on an Illumina platform yielded 11.1 Gb of data. Assembly with Trinity produced 150 795 contigs and a BUSCO completeness of 94.1%. Because a previous study assembled RNA from several life cycle stages and obtained approximately 770 k contigs [23], the smaller contig number recovered in our work, which was based only on two plasmodial conditions, is considered reasonable. Approximately 50% of the contigs received UniProt-based functional annotations. Contamination screening by DIAMOND BLASTX against UniProt reference proteomes and visualization with BlobTools revealed no GC-content clusters indicative of foreign sequences (figure 2*c*).

### (c) Differential gene expression analysis

To elucidate the intracellular responses in *P. roseum*, we cultured plasmodia under four combinations of light (L, light; D, dark) and nutrient (S, starvation; N, nutrient-rich) conditions: DS, LS, DN and LN. Triplicate RNA-seq libraries (*n* = 3) were prepared for each condition, generating 10.5−30.7 million reads per sample that were mapped to the *P. roseum* reference transcriptome.

The mapping rates for all 12 libraries were all above 90%, with average values of 93.1% for DN, 98.6% for DS, 95.2% for LN and 95.4% for LS. These high matching rates suggest that the de novo transcriptome assembly comprehensively captured expressed transcripts. Therefore, the analysis below will focus on the results of the gene expression variation analysis and their biological implications.

#### (i) Global expression patterns

Spearman rank correlations revealed that the DN condition—standard plasmodial maintenance—shared only modest similarity (*ρ* ≒ 0.7) with each of the other three conditions, whereas DS, LS and LN were more closely related to one another (*ρ* = 0.8−0.9). Thus, both light exposure and nutrient depletion provide stimuli sufficient to shift the overall transcriptional profile away from that of the standard.

#### (ii) Differentially expressed genes counts

**Figure 3.**
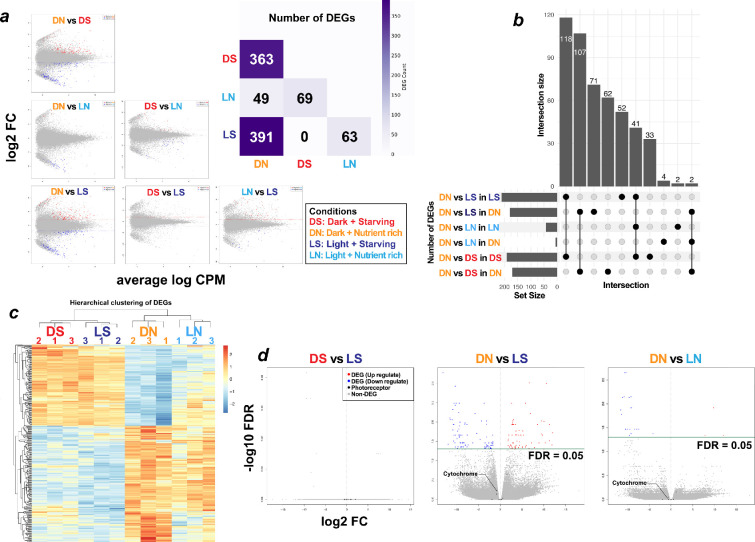
Results of differential gene expression analysis of *Physarum roseum*. (*a*) DEG counts (FDR < 0.05, |log₂FC|>2) and MA plots (*y*-axis: log₂ fold-change; *x*-axis: mean log expression) for each condition. The grey plots indicate genes that are not DEGs. Relative to the dark, nutrient-rich baseline (DN), significant DEGs were detected in all other conditions, with the largest numbers arising from nutrient-deprived treatments. Light–dark comparisons (L versus D) yielded only limited DEG sets, indicating that most transcriptional changes are driven by nutrient availability (N versus S) rather than illumination. (*b*) UpSet plot of DEG overlap. Light–dark contrasts (L versus D) produce few DEGs and minimal overlap, whereas nutrient contrasts (N versus S) share extensive sets of consistently up- or downregulated genes. A small subset was upregulated in every comparison except the DN baseline, underscoring nutrient-dominated transcriptional programming. (*c*) Hierarchical clustering of DEGs. The samples were clustered cleanly according to the experimental conditions, with nutrient availability emerging as the primary driver of grouping. (*d*) Volcano plot (light versus dark): red = light upregulated genes; blue = dark upregulated; black = photoreceptor genes (isoforms collapsed). InterProScan-labelled photoreceptor domains are named, but none exceed the DEG threshold and most change only slightly.

We quantified differentially expressed genes (DEGs) to measure their impact on the overall transcriptional profile. From the DN condition, nutrient starvation produced >300 DEGs regardless of light (figure 3*a*), whereas light and dark contrasts yielded only a few to none. In a previous study, *P. polycephalum* displayed 6985 light-responsive DEGs [24], highlighting *P. roseum*’s marked light insensitivity. Although starvation induced more DEGs than light in *P. roseum*, the scale remained far below the system-wide shifts reported during sporulation in earlier studies.

#### (iii) Nutrient-driven signature

We next compared functional overlap among DEGs when the standard DN culture conditions were perturbed either by illumination or nutrient withdrawal. Focusing on the two contrasts that yielded the largest DEG sets—DN versus LS and DN versus DS—more than 100 genes were commonly upregulated by starvation regardless of light regime. Similarly, more than 100 genes were downregulated under the same conditions, meaning that roughly two-thirds of all DEGs were shared between the two comparisons (figure 3*b*). In other words, once *P. roseum* experiences any perturbation of the usual culture conditions, the vast majority of expression changes are driven by nutrient status, whereas light responsiveness remains a minority component.

This pattern was mirrored in the expression-level clustering analysis. Hierarchical clustering of DEGs expressed in all samples (TPM > 0) revealed that the largest clade segregated strictly by nutrient availability (figure 3*c*). No clusters were intermixed across environmental conditions, further demonstrating that nutrient deprivation exerts a far stronger influence on the transcriptome of *P. roseum* than does the light–dark regime.

#### (iv) Photoreceptor genes

Previous studies on *P. polycephalum* have identified two classes of photoreceptor-related genes: (i) a starvation-induced, phytochrome-like photoreceptor (*phyA, phyB*) [6,23] and (ii) three genes that are constitutively expressed and were identified in the genome—the LOV (Light–Oxygen–Voltage) domain photoreceptor LovA, the cryptochrome cryA and a DNA photolyase (plyA) [21]. Accordingly, we investigated whether the light-insensitive *P. roseum* exhibits comparable photoreceptor behaviour. First, none of the five photoreceptor genes reported in *P. polycephalum* were expressed under any of the conditions tested. To identify function-based homologues, we predicted domains for the five candidate genes with InterProScan and queried those domains against the Pfam annotations assigned to our de novo transcriptome assembly. For each of the five genes, we detected a single transcript that possessed an identical photoreceptor-related domain. However, none of these transcripts were classified as DEGs under any condition, and all displayed TPM values less than 10. These findings suggest that these genes had low expression and were unlikely to be functionally active. Second, to cast a wider net for photoreceptor-related genes in *P. roseum*, we conducted a GO term-based search. As a result, 92 photoreceptor-related genes (including isoforms) were retrieved; however, most were not expressed, and only 13 exhibited appreciable TPM values. Among these genes, six genes were highly expressed (≥10th percentile) across all conditions. Nevertheless, every one of the 13 genes displayed only minor expression shifts between conditions (|log₂FC| < 2), and no significant DEGs were detected (figure 3*d*).

These results indicate that *P. roseum* responds to starvation but lacks the photoreceptor induction, light-triggered sporulation and transcriptomic shifts seen in *P. polycephalum*. The absence of light-dependent DEGs, despite constitutive photoreceptor genes, implies these receptors are largely inactive. Thus, *P. roseum* is markedly less light-responsive, indicating weak conservation of the sporulation network within *Physarum*.

## 4. Discussion

### (a) Evaluation of the de novo transcriptome assembly

In this study, we generated a de novo transcriptome for *P. roseum* from field-collected plasmodia using simple mechanical disruption and column purification. Even with this minimal protocol, the assembly was highly complete (BUSCO 94.1%), showing that fresh plasmodia can provide a reliable molecular reference without axenic culture. BLAST screening marked several contigs as Proteobacteria (figure 2*c*), removing them lowered BUSCO completeness to 90.6%. Because prokaryotes rarely carry eukaryotic core genes, this drop likely reflects horizontal gene transfer rather than misassembly or large-scale contamination—especially given the poly-A selection.

This approach provides a quick, convenient path to new molecular references for slime moulds. Because most myxomycetes still lack reference sequences, our study serves as a proof of concept for generating practical transcriptomes with minimal effort.

### (b) Comparison of the responses of three *Physarum* species to starvation + light

Previous studies have shown that *P. polycephalum* sporulates almost invariably after 3 days of starvation followed by light exposure [7,19]. Under the same regime, we observed no sporulation in *P. roseum*, whereas *P. rigidum* sporulated in four of the six plasmodia (figure 1). These results demonstrate pronounced interspecific differences in physiological responses within the genus: *P. rigidum* behaves more like *P. polycephalum*, whereas *P. roseum* is markedly refractory. Although *P. rigidum* has not yet been included in molecular phylogenetic studies, its ecological similarity to *P. polycephalum*—especially in terms of sporulation and culture behaviour—suggests a closer relationship than that of *P. roseum*.

Focusing on the non-responsive species, we analysed the transcriptomic dynamics of *P. roseum* under four conditions—light (L) or dark (D) and nutrient-rich (N) or starvation (S). Field plasmodia showed within-condition correlations of *r* = 0.8−0.9, slightly lower than axenic cultures yet sufficient for DEG detection. From the DN baseline, every perturbation altered expression but far less than the dramatic shifts that accompany sporulation in *P. polycephalum* (figure 3*a*). Light alone produced few DEGs, and starvation failed to induce the phytochrome-like photoreceptors or downstream cascade characteristic of *P. polycephalum* [24]. Although both species possess constitutive photoreceptor genes, their repertoires differ; in *P. roseum,* these genes are weakly expressed and show no light-dependent regulation (figure 3*d*), indicating they play little role in its sporulation. Furthermore, photoreceptor-related genes implicated in sporulation in *P. polycephalum* were not confirmed in *P. roseum*, and no transcripts with matching domains were expressed, indicating that a comparable response system was absent. One possible reason is that the transcriptome reference was generated under less varied conditions than in previous studies. However, the high RNA-seq mapping rate shows that, at least in *P. roseum*, these photoreceptor genes were scarcely expressed under light and starvation stimuli. Future work should employ structural genomics and qPCR validation to determine whether these genes are conserved and to clarify their functions.

The findings above demonstrate that *P. roseum* exhibits far fewer responses to environmental cues than *P. polycephalum* and is especially robust to light stimuli. Unlike *P. polycephalum, P. roseum* seems to cope with environmental changes, even harsh ones such as sudden illumination or prolonged starvation, by remaining in the plasmodial state as long as possible.

Although we were unable to induce sporulation in *P. roseum* under our laboratory regime, fruiting bodies have been observed in nature. Two possibilities, therefore, arise. First, the starvation treatment used here may have been insufficient as a preconditioning step; in the field, complete starvation for 3 days or more is unlikely for a predatory plasmodium. Second, additional cues beyond starvation and light—such as pH, humidity, or, most plausibly, temperature—may be needed. Moreover, natural environments show considerable variability in both nutrient availability and lighting conditions. Our laboratory conditions may not completely replicate the complexity of cues necessary to trigger sporulation in *P. roseum. P. roseum* is frequently reported in warm regions of Asia, Central America and South America [18,25,26], and most isolates, including the one used in this study, were collected in summer. In contrast, *P. polycephalum* is typically cultured and induced to sporulate at 20−25°C. *P. roseum* may, therefore, require relatively high temperatures in addition to the broadly conserved starvation-and-light stimuli. In addition to environmental factors, the state of the plasmodium may also influence the trigger of sporulation. Several myxomycete species producing large aggregations of fruiting bodies in nature. If *P. roseum* exhibits similar behaviour, reaching a plasmodial size beyond a certain threshold could serve as a trigger.

Such weak conservation of a supposedly fundamental sporulation response exceeds previous expectations and will be important for future work on the diversity and systematics of myxomycetes. Molecular phylogenetic analysis using marker regions in previous studies has shown that *P. polycephalum* and *P. roseum* belong to different genera [14], and the low degree of functional conservation observed here supports this conclusion. However, genomic information from multiple species will be necessary to perform molecular phylogenetic analysis that incorporates functional information. In *P. polycephalum*, it will also be necessary to elucidate the details of the specific systems in which photoreceptor genes are involved in sporulation.

Notably, field discoveries of *P. polycephalum* are exceedingly rare [27], whereas *P. roseum* appears to be widespread and stable—perhaps owing to the environmental robustness revealed herein. Although *P. polycephalum* remains the canonical model species because its culture conditions are well defined and molecular resources are well developed, its limited field availability has begun to hamper studies that require multiple isolates [28]. From the standpoint of versatility and environmental tolerance, the continued reliance on *P. polycephalum* as the sole model may be questionable. With further refinement of culture methods and the acquisition of genomic references, *P. roseum* and other species could well emerge as more suitable model organisms for future molecular research.

## Data Availability

The raw data used in this research are available from PRJNA1253572. Spectrum data of light conditions are available from the Dryad Digital Repository [29]. Supplementary material is available online [30].
